# Effect of TGF-β1 on the Migration and Recruitment of Mesenchymal Stem Cells after Vascular Balloon Injury: Involvement of Matrix Metalloproteinase-14

**DOI:** 10.1038/srep21176

**Published:** 2016-02-16

**Authors:** Wei Zhao, Chengyan Wang, Ruixue Liu, Cuilei Wei, Juncang Duan, Kejian Liu, Shugang Li, Hong Zou, Jin Zhao, Lianghai Wang, Yan Qi, Weihua Liang, Jinfang Jiang, Wenjie Zhang, Lijuan Pang, Feng Li

**Affiliations:** 1 Department of Pathology and Key Laboratory of Xinjiang Endemic and Ethnic Diseases (Ministry of Education), Shihezi University School of Medicine, 59 North 2nd Road, Shihezi, Xinjiang 832002, China; 2 Department of Cardiology, the First Affiliated Hospital, Shihezi University School of Medicine, 45 North 3rd Road, Shihezi, Xinjiang 832008, China; 3 Department of Public Health, Shihezi University School of Medicine, 59 North 2nd Road, Shihezi, Xinjiang 832002, China

## Abstract

Restenosis or occlusion after vascular procedures is ascribed to intimal hyperplasia. Transforming growth factor (TGF)-β1 is involved in recruitment of mesenchymal stem cells (MSCs) following arterial injury, and its release from latent TGF-binding protein by matrix metalloproteinase (MMP)-14-induced proteolysis contributes to neointima formation. However, the relationship between MMP-14 and TGF-β1 activation in restenosis is unknown. This study investigated the relationship using a rat model of balloon-induced injury. Rats were assigned to vehicle-, SB431542 (SB)-, or recombinant human (rh)TGF-β1-treated groups and examined at various time points after balloon-induced injury for expression of TGF-β1/Smad signalling pathway components, MMP-14 and MSCs markers including Nestin, CD29, and Sca1^+^CD29^+^CD11b/c^−^CD45^−^. Intimal hyperplasia was reduced in SB- and rhTGF-β1-treated rats. The expression of TGF-β1, TGF-β1RI, and Smad2/3 was decreased, but the levels of phosphorylated Smad2/3 were higher in SB-treated rats than vehicle-treated after 7 days to 14 days. rhTGF-β1 administration decreased the expression of TGF-β1/Smad pathway proteins, except for TGF-β1RI. Nestin and CD29 expression and the number of Sca1^+^CD29^+^CD11b^−^CD45^−^ cells were reduced, whereas MMP-14 expression was increased after SB431542 and rhTGF-β1 administration. These results suggest that TGF-β1/Smad signalling and MMP-14 act to recruit MSCs which differentiate to vascular smooth muscle cells and mesenchymal-like cells that participate in arterial repair/remodelling.

Coronary heart disease (CHD) is the most common cause of death worldwide^1^. In a hospital setting, various investigative procedures are used to diagnose CHD and determine the need for coronary revascularisation. Percutaneous transluminal coronary angioplasty (PTCA) is the most effective means of revascularisation, however, incomplete re-endothelialisation and neointimal hyperplasia limitits long-term efficacy^2^,^3^,^4^. The mechanism underlying neointimal hyperplasia is unclear, although animal and clinical studies have shown that restenosis is related to intimal hyperplasia and vascular remodelling following PTCA^5^,^6^,^7^.

The origin and function of cells involved in neointimal formation are not known. It is widely acknowledged that vascular smooth muscle cells (VSMC) are activated after arterial injury and induce neointimal formation through migration and proliferation^8^. Recent studies, however, have demonstrated that vascular adventitial cell migration is the main mechanism of intimal hyperplasia^9^,^10^. In addition, hematopoietic stem cells and mesenchymal stem cells (MSCs) are mobilised from the bone marrow and recruited to sites of injury where they contribute to tissue remodelling^11^.

Transforming growth factor (TGF)-β1 is involved in various physiological processes including the regulation of cell proliferation, differentiation, migration, and extracellular matrix production, for instance, in cardiovascular development^12^. TGF-β1 also plays an essential role in the pathogenesis of restenosis^13^ and is expressed at high levels in intimal hyperplastic lesions^14^. MSCs transplantation after vessel injury has demonstrated its contribution to neointimal formation^15^. Therefore, evaluating the role of activated TGF-β1 in the migration and differentiation of MSCs may provide insights into the repair and remodelling of cardiac tissue.

Extracellular matrix (ECM) molecules play an important role in restenosis after intimal injury^16^ by providing a supportive matrix scaffold for cells and supplying growth factors. Matrix metalloproteinases (MMPs) can degrade various components of the ECM and mediate ECM remodelling in physiological and pathological processes. MMP-14 (also known as MT1-MMP) is a membrane-associated protein that participates in vascular remodelling by promoting the migration and proliferation of VSMC^17^,^18^ and regulates MSCs migration, invasion, and proliferation^19^,^20^. Moreover, MMP-14 has been shown to release latent TGF-β1 from latent TGF-binding protein (LTBP)^21^. Understanding the relationship between TGF-β1 and MMP-14 and their potential roles in MSCs recruitment can provide important insights into the mechanism underlying restenosis and neointimal formation.

In this study, the expressed components of TGF-β/Smad signalling pathway and MMP-14 and their effects on MSCs in neointimal formation have been examined in a rat model that has left carotid arterial balloon injury by administrating inhibitor of TGF-β1 superfamily type I activin receptor-like kinase (ALK) receptor SB431542 (SB) or recombinant human (rh)TGF-β1. The findings presented are novel and may shed light on a possible role of TGF-β1 in cardiac remodelling, which has the potential in the prevention and/or treatment of post-operative restenosis in humans.

## Results

### Histological and morphometric analyses of neointima

**Figure 1:**
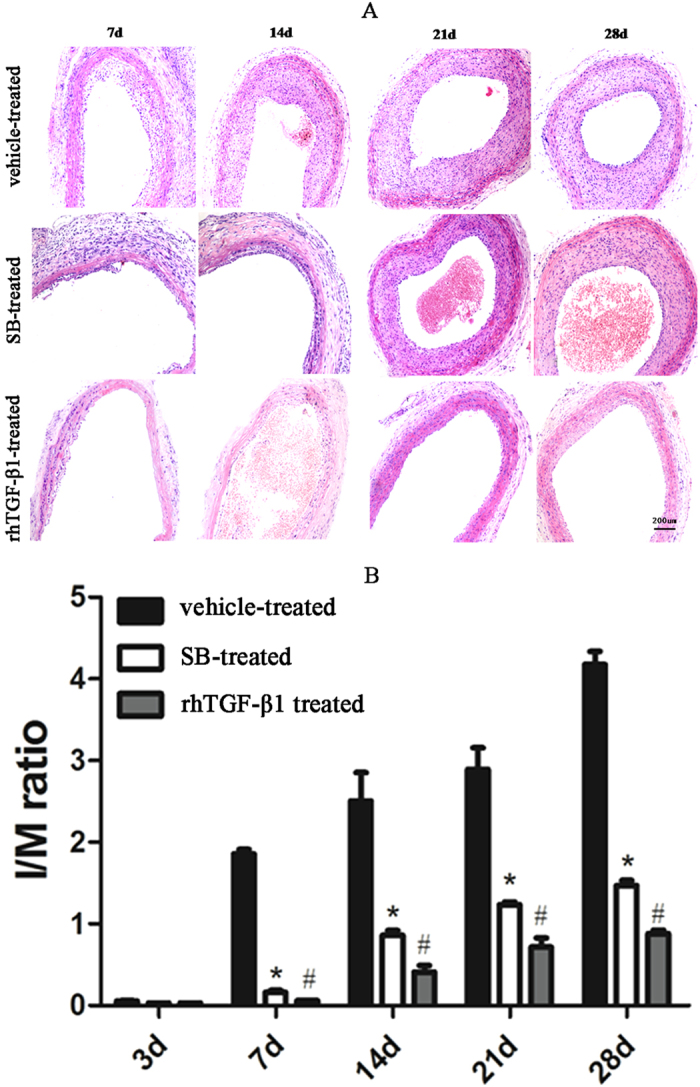
Neointimal thickness and degree of hyperplasia reduced by TβRI/Smad inhibitor and rhTGF-β1 in response to arterial remodelling. (**A**) Haematoxylin and eosin staining of injured carotid arteries from rats treated with vehicle, SB431542 (SB)or rhTGF-β1. Scale bar, 200 μm. (**B**) I/M ratios. Results represent mean ± SD (n = 5 per group). *P < 0.05, SB-vs vehicle-treated group; ^#^P < 0.05,rhTGF-β1- vs vehicle-treated group.

Intimal growth occurred at different time points after injury. Neointima was barely formed at 0 h, 8 h, 1 day and 3 days in injured arteries. However, Neointima was visible at 7 days with maximal thickness at 28 days after injury (Fig. 1A). In contrast, neointima was markedly thinner in rats treated with 0.2 mg SB431542 or 40 ng rhTGF-β1. The degree of neointimal hyperplasia was assessed by intima/media (I/M) ratio. Ratios were lower in the SB- and rhTGF-β1-treatedrat group than in the vehicle-treated rat group (P < 0.05) (Fig. 1A,B). These results demonstrated that inhibitor of TGF-β1 superfamily type I activin receptor-like kinase receptor blocked neointimal formation and vascular restenosis after arterial injury and that rhTGF-β1 treatment did not induce neointimal formation.

### Activation of TGF-β/Smad signalling pathway is essential for arterial remodelling in response to injury

**Figure 2:**
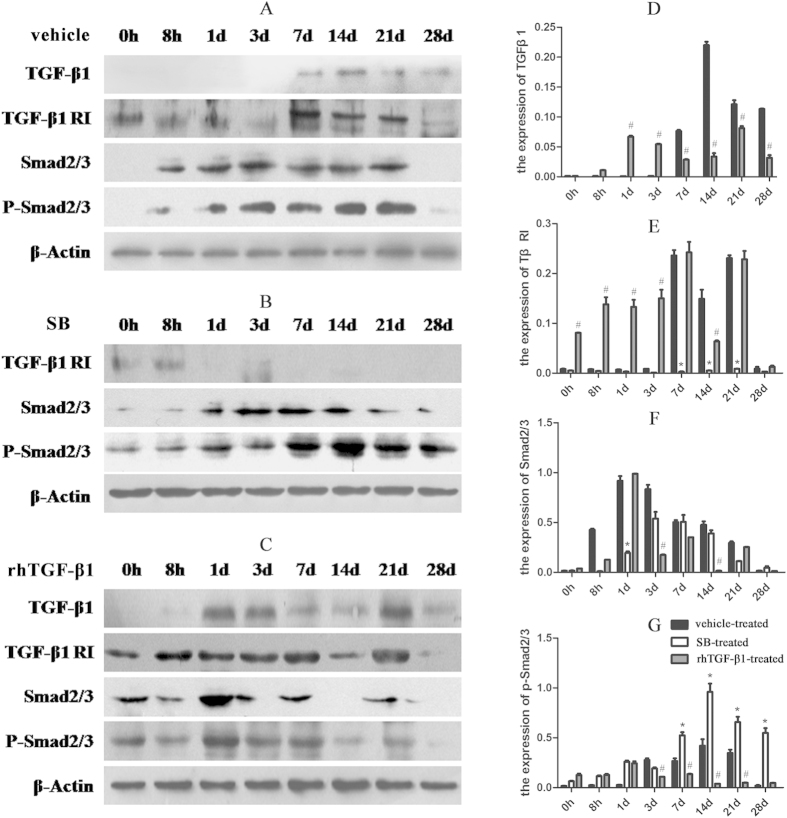
Expression levels of TGF-β1/Smad signalling proteins after arterial injury. (**A**–**C**) Expression levels of TGF-β1 (12.5–25 kDa), TβRI (53 kDa), Smad2/3 (48 kDa), and P-Smad2/3 (52 kDa) after balloon angioplasty in rats treated with vehicle (**A**), SB431542 (**B**), or rhTGF-β1 (**C**), as detected by western blotting. The blots were cropped and the gels were run under the same experimental conditions. (**D**–**G**) Densitometry analysis of TGF-β1 (**D**), TβRI(**E**), Smad2/3 (**F**), and P-Smad2/3 (n = 5 per group per experiment). *P < 0.05, SB-vs vehicle-treated group; ^#^P < 0.05, rhTGF-β1 vs vehicle-treated group.

**Figure 3:**
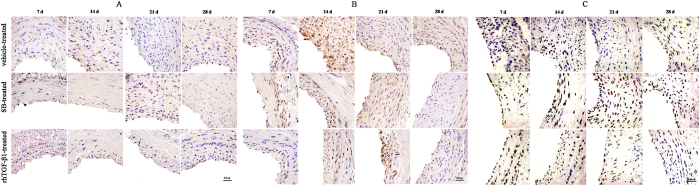
Immunohistochemical analysis of TGF-β1/Smad signalling protein expression in injured arteries. (**A**–**C**) Representative images of TGF-β1 (**A**), TβRI (**B**), and P-Smad2/3 (**C**) expression. Scale bar, 50 μm.

The expression of TGF-β1, TβRI, and phosphorylated (P-)Smad2/3 was evaluated by western blotting and immunohistochemistry (Figs 2 and 3). In the vehicle-treated group, TGF-β1 level increased on day 7, reaching a maximum day 14, before decreasing on day 21 and 28 (Fig. 2A). TGF-β1 activation was not observed in the SB-treated group(Fig. 2B), TGF-β1 level decreased in the rhTGF-β1-treated group at 7, 14, 21, and 28 days (Fig. 2C,D). TβRI expression decreased relative to the vehicle-treated group (Fig. 2B,E) after treatment with SB431542 but was increased in the rhTGF-β1-treated as compared vehicle-treated group (Fig. 2C,E). Smad2/3 level was elevated at 8 h, reaching a maximum on day 1 and decreasing up to day 28 in the vehicle-treated group(Fig. 2A). In SB-treated animals, Smad2/3 expression was increased on day 1, peaked on day 14, and decreased by day 21(Fig. 2B). Administration of rhTGF-β1 induced an increase in the expression of Smad2/3 on day 1, followed by a decrease on day 3 (Fig. 2C,F). Furthermore, P-Smad2/3 level was lower in SB-treated than in vehicle-treated animals before 3 days, but after 7 days to 14 days, the expression of P-Smad2/3 was increased after injecting with SB (Fig. 2A,B and G). but it was downregulated in the rhTGF-β1-treated group(Fig. 2C,G). Immunohistochemical analysis revealed that TGF-β1, TβRI, and P-Smad2/3 were localised to the neointima (Fig. 3A–C).

### MSCs enters the peripheral blood in response to vascular injury

**Figure 4:**
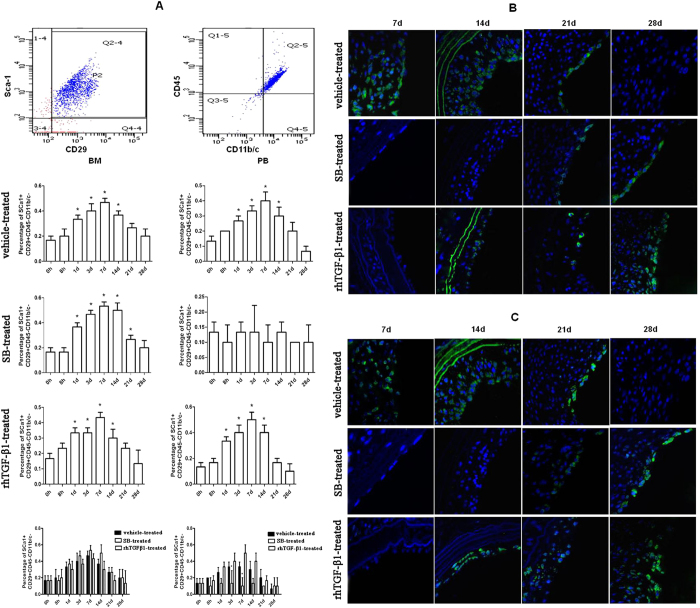
Injury-activated TGF-β1 induces the mobilisation of MSCs for tissue repair. (**A**) Percentages of Sca1^ + ^CD29^ + ^CD11b^−^CD45^−^ cells in peripheral blood and bone marrow at indicated time points in injured rats treated with vehicle, SB431542 (SB), and rhTGF-β1. Results represent mean ± SD (n = 5 per group per time point). *P < 0.05, SB- vs. vehicle-treated rats. (**B**,**C**) Immunofluorescence analysis of Nestin (**B**) and CD29 (**C**) expression in vehicle-, SB-, and rhTGF-β1-treated injured rats. Scale bar, 50 μm.

The mobilisation of MSCs from bone marrow to peripheral blood is a prerequisite for tissue repair/remodelling. This study aimed to assess whether Sca1^+^CD29^+^CD11b/c^−^CD45^−^MSCs was induced to proliferate, mobilize in peripheral blood, and be recruited to sites of damaged vessels in rats with common carotid artery injury. The number of Sca1^+^CD29^+^CD11b/c^−^CD45^−^ cells in peripheral blood and bone marrow were elevated 1 day after injury in the vehicle-treated rat group, which lasted for 14 days. SB-treatment decreased the number of Sca1^+^CD29^+^CD11b/c^−^CD45^−^ cells in peripheral blood, however, the number of Sca1^+^CD29^+^CD11b/c^−^CD45^−^ cells in the bone marrow, was increased 1 day post-injury and remained elevated for 21 days. In rhTGF-β1-treated rats, the number of Sca1^+^CD29^+^CD11b/c^−^CD45^−^ cells was increased in both the peripheral blood and bone marrow 1 day post-injury, which lasted for 14 days. Although there was no difference in the number of Sca1^+^CD29^+^CD11b/c^−^CD45^−^ cells in the bone marrow among the three rat groups, the number of Sca1^+^CD29^+^CD11b/c^−^CD45^−^ cells in the peripheral blood was decreased in the SB-treated rats but not in the rhTGF-β1-treated rats on days 3 and 7 as compared with rats in the vehicle-treatment group (Fig. 4A).

### MSCs are recruited to remodel arteries in response to vascular injury

The expression of CD29 and Nestin was examined by immunofluorescence in order to determine the localisation of recruited MSCs. Nestin- and CD29-positive cells were present on the luminal side of neointimal tissue. In vehicle-treated rats with common carotid artery injury, more Nestin-positive cells were observed on day 14 than on days 7 and 21, while no positive cells were detected at other time points. In SB-treated injured rats, there were a few positive cells on days 21 and 28 but none at other time points. In rhTGF-β1-treated rats, there were more positive cells on day 28 than on days 14 and 21 but none at other time points (Fig. 4B). A correlation between the numbers of CD29- and Nestin-positive cells was observed on the luminal side of the neointimal tissue (Fig. 4C).

**Figure 5:**
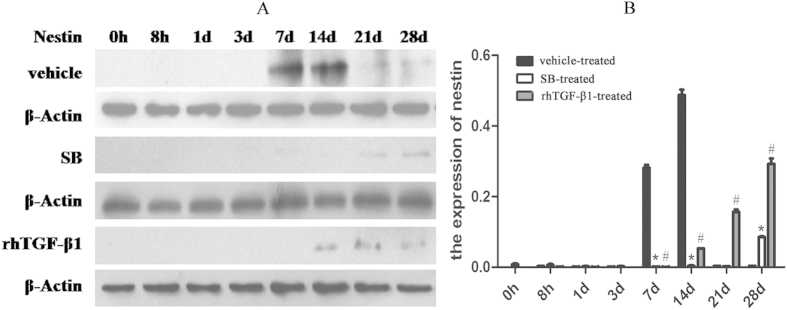
Nestin expression after arterial injury. Western blot analysis of Nestin(177 kDa) expression in vehicle-, SB431542 (SB)-, and rhTGF-β1-treated rats with common carotid arterial injury. *P < 0.05, SB- vs. vehicle-treated rats; ^#^P < 0.05, rhTGF-β1- vs. vehicle-treated rats. The blots were cropped and the gels were run under the same experimental conditions.

To determine whether MSCs accumulated in the injured artery, Nestin levels were evaluated by Western blotting. In vehicle-treated rats, Nestin expression was upregulated on day 7 and to a greater extent on day 14; in SB-treated group, the expression was increased on day 28. In rhTGF-β1-treated rats, Nestin was highly expressed on day 28 relative to days 14 and 21, while no expression was detected on day 7. Nestin expression levels differed significantly between SB- and rhTGF-β1-treated rats with respect to vehicle-treated rats on days 7, 14, 21, and 28 (Fig. 5A,B).

### Contribution of endothelial cells to vascular repair

The expression of endothelial cell markers, CD31 and CD34, was detected by immunohistochemistry. CD31- and CD34-positive cells were detected in the neointima in all three groups (Supplementary Fig. 1B and Supplementary Fig. 1C). Meanwhile, the expression of Ki-67, a marker reflecting cell proliferation, in neointimal cells peaked at 14 days, but decreased after administering SB or rhTGF-β1 as compared with vehicle-treated rats (Supplementary Fig. 1A).

### VSMC- and mesenchymal-like phenotype cells in neointimal cells

**Figure 6:**
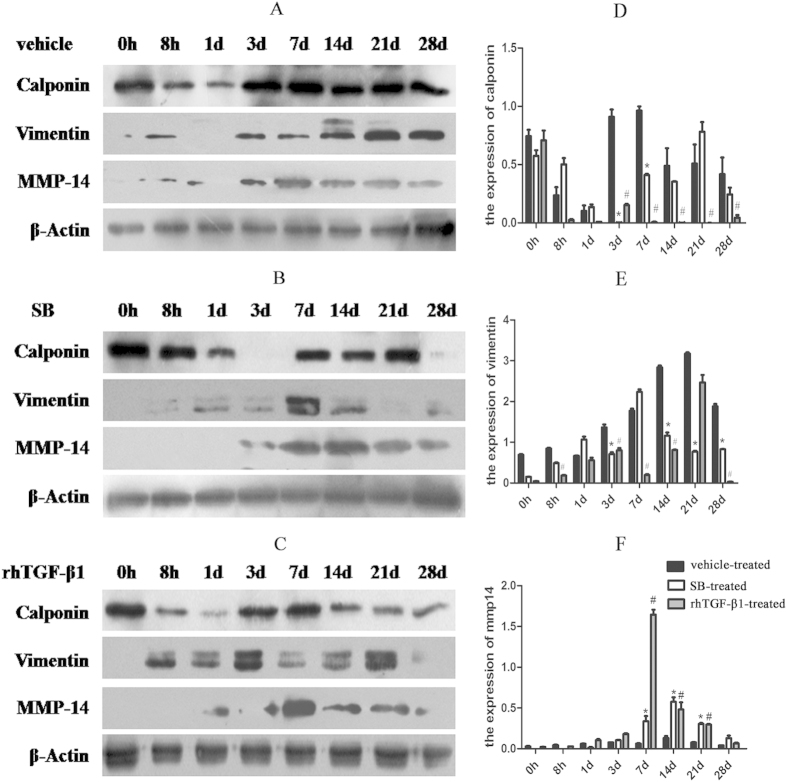
Calponin, SMA, and Vimentin expression after arterial injury. (**A**–**C**) Expression of Calponin (34 kDa), Vimentin (57 kDa), and MMP-14 (66 kDa) after balloon angioplasty in rats treated with vehicle (**A**), SB431542 (SB) (**B**), and rhTGF-β1, as detected by western blotting. The blots were cropped and the gels were run under the same experimental conditions. (**D**–**F**) Densitometry analysis of Calponin (**D**), Vimentin (**E**), and MMP-14 (**F**) expression (n = 5 per group per experiment). *P < 0.05, SB- vs. vehicle-treated group; ^#^P < 0.05, rhTGF-β1- vs. vehicle-treated group.

To determine the cell type associated with the neointima and evaluate the role of MSCs in neointima formation, Calponin, Smooth Muscle Actin (SMA), and Vimentin expression was examined by western blotting and immunohistochemistry. In vehicle-treated rats, Calponin level increased on day 3, peaked on day 7, and decreased thereafter (Fig. 6A); in the SB-treatment group, Calponin was upregulated on day 7 and reached a maximum on day 21 (Fig. 6B); and in rats treated with rhTGF-β1, the level decreased at 14, 21, and 28 days (Fig. 6C). Calponin-positive cells were located at the cell cytoplasm of the neointima, as detected by immunohistochemistry (Supplementary Fig. 2A). SMA level increased at 7, 14, 21, and 28 days in vehicle-treated rats, but declined after administration of SB431542 or rhTGF-β1 (Supplementary Fig. 2B). Vimentin expression was upregulated on day 3 and peaked on day 21 in vehicle-treated rats (Fig. 6A), but was downregulated in SB- and rhTGF-β1-treated rat groups (Fig. 6B,C,E).

### Role of MMP-14 in neointimal formation

To examine the correlation between MMP-14 and TGF-β1 in vascular tissue repair, MMP-14 expression was assessed by Western blotting and immunohistochemistry. In the vehicle-treated rat group, MMP-14 expression was increased at 3 days, peaked at 14 days, and then decreased by day 28 after injury (Fig. 6A). In rats treated with SB or rhTGF-β1, the level of MMP-14 was increased at 7 days, and in the SB-treated group, the increased level of MMP-14 persisted at 14, 21, and 28 days (Fig. 6B,F). MMP-14 was distributed within the neointima of the carotid artery, with positive immunoreactivity observed in the cytoplasm of neointima cells (Supplementary Fig. 3).

## Discussion

Despite current medical treatments and improved survival rates, CHD remains the leading cause of death worldwide, especially in developed countries^22^. In China, the number of CHD cases has been rapidly increasing in recent years. PTCA is frequently used for treatment but its efficacy is often limited by the development of restenosis resulted from neointimal formation^23^, which is implicated in angioplasty, stent restenosis, and transplant vasculopathy^24^,^25^. This problem can only be alleviated through a better understanding of the underlying cellular and molecular mechanisms.

TGF-β is an important regulator of vascular remodelling, including atherosclerosis, restenosis, and hypertension. Each of its three isoforms (β1, β2 and β3) has a distinct function that is exerted via different receptors. TGF-β mRNA and protein levels are upregulated during neointimal thickening following arterial injury^26^,^27^. TGF-β1 plays an essential role in wound healing and tissue remodelling^28^,^29^,^30^,^31^,^32^and is mainly expressed in the cardiovascular system, where it stimulates vascular endothelial cell and fibroblast proliferation as well as regulates the secretion and increases the adhesiveness of the extracellular matrix. We used the TGF-β1 inhibitor, SB431542, to block the activation of TGF-β1 signalling pathway and found that neointimal formation was inhibited and the degree of hyperplasia was reduced. Administration of SB431542 inhibited the expression of TGF-β1, TβRI, and Smad2/3, which was upregulated after injury. As a key step, the phosphorylation of Smad2/3 represents the activation of Smad signalling pathway^33^, which can be suppressed as shown in studies by Kim and Xu^34^,^35^. In their studies, blockage of TGF-β1 signaling using SB431542 significantly reduced Smad3 phosphorylation and expression.We suspected that the expressed level of P-Smad 2/3 would be also reduced in SB-treated group, in which SB431542 played a role until 3 days in the rat arterial injury model, but after 7 days to 14 days, the expression of P-Smad2/3 was increased after injecting with SB431542. We assumed that there would be other molecules involved in the phosphorylation of Smad2/3. In the early stage of injury, TGF-β1/Smad signaling pathway may play a leading role. Nevertheless, other molecules could also participate in activating Smad proteins besides TGF-β in the middle and/or late stages of arterial injury. TGF-β1/Smad pathway may play a role in artery injuries in a time-dependent manner. Kim BG *et al.*^36^ have demonstrated that bone morphogenetic protein-2 (BMP-2) can also phosphorylate the C terminus of Smad2 and they propose that both TGF-β1 and BMP-2 signaling can regulate Smad pathways. SB-431542 is not only a selective inhibitor of endogenous activin but also has no apparent effect on BMP signaling. The expression of P-Smad2/3 was increased after injecting with SB431542 after 7 days to 14 days in our animal model, BMP signaling may play the dominant role. It needs further investigate. Meanwhile, an article^37^ published in Circulation has reported that growth-arrested VSMCs treated with Angiotensin II (Ang II) for 20 minutes, the expression of phosphorylated Smad2 protein level was increased, and they proposed that TGF-β blockade did not modify Ang II–induced activation of Smad pathway, suggesting a TGF-β–independent Smad signaling elicited by Ang II. Moreover, Kido *et al.*^38^ have confirmed that mechanical stress could cause increased phosphorylation of Smad2/3. A study by Gerhild Euler-Taimor and Jacqueline Heger has come to a similar conclusion in which they propose that Smad proteins are not only activated by TGF-β but also associated with other signaling pathways^39^. Apparently, further studies are required to elucidate whether Smad2/3 is able to be phosphorylated by other signaling pathways. In rats treated with rhTGF-β1, neointima formation was barely visible at any time point, contrary to our prediction. This may be due to the route of administration; that is, with a local injection, TGF-β1 remains concentrated at the site of injury. In addition, exogenous TGF-β1 may inhibit differentiation by blocking cell cycle progression^40^, which may have abolished the TGF-β1 concentration gradient.

Recently studies have demonstrated that MSCs can promote tissue repair after injury^41^. MSCs are first identified in the bone marrow as a colony-forming cells with osteogenic, chondrogenic, and adipogenic potential^42^. MSCs are implicated in many cellular processes owing to their capacity for self-renewal, proliferation, migration, and invasion^43^,^44^,^45^. Following injury and inflammation, MSCs promote tissue regeneration via regulating cell proliferation and differentiation^46^,^47^. TGF-β1 recruits MSCs to the injury site for bone repair/reconstruction^48^. We found that the number MSCs of Sca1 + CD29 + CD11b/c − CD45− which are the markers of MSCs^49^,^50^ was elevated in the peripheral blood and bone marrow starting 1 day after injury and lasting up to 14 days. In addition, neointima cells expressed high levels of MSC markers Nestin^51^ and CD29^16^. In rats treated with SB431542, the Sca1 + CD29 + CD11b/c − CD45− cell population was increased in the bone marrow but was not detectable in the peripheral blood, indicating that TGF-β1 inhibition blocked the release of bone marrow MSCs into peripheral blood for tissue remodelling. The number of Nestin- and CD29-positive cells was decreased in the lumen of injured arteries, but the injection of rhTGF-β1 induced an increase in the number of Sca1 + CD29 + CD11b/c − CD45− cells in the peripheral blood and bone marrow that persisted for 14 days. The decrease in the number of Nestin- and CD29-positive cells at 7, 14, and 21days may be due to the loss of the TGF-β1 concentration gradient. Our results showed that on day 7, MSCs were recruited to the injured site where they contributed to the neointimal formation until day 21. In a wire-induced vascular injury model, MSCs adhered to injured vessels and showed a strong capacity for differentiation into neointimal cells^52^,^53^. Our results suggested that TGF-β1 recruited MSCs to the injury site via the chemotactic mechanism and stimulated their proliferation for tissue remodelling.

TGF-β1 can induce cellular transformation^54^. It has been shown to inhibit VSMC proliferation under normal circumstances as well as in response to vascular injury^55^. We observed that Calponin and SMA levels increased at 7 days after injury and such increased levels persisted up to 28 days. Treatment with SB431542 resulted in a decrease in the expression of these factors, consistent with a previous report that examined smooth muscle cells^56^. Similarly, Vimentin level was at maximal level at 14 and 21 days, but decreased upon treatment with SB431542 or rhTGF-β1, in keeping with previous findings that blocking TGF-β activation was able to suppress Vimentin expression^57^. These findings confirm that TGF-β signalling participates in neointimal formation by inducing a VSMC-like and mesenchymal-like cellular phenotype. We also observed weak expression of endothelial markers CD31 and CD34 in all rats, suggesting that endothelial cells may differentiate into other cell types at 7 days after injury.

The ECM plays an important role in vascular development and morphogenesis and the interaction between vascular cells and the ECM is critical in blood vessel formation and remodelling. MMPs contribute to these processes by degrading ECM so as to facilitate cell migration and invasion^58^. Both vascular and inflammatory cells in vessel walls produce MMPs and a reduction in MMP-14 expression has been shown to repair damaged vessel walls *in vivo*^59^. Overexpressed MMP-14 enhances MSCs migration and invasion^60^, which is required in angiogenesis^61^. We found increased levels of MMP-14 at 3 days after injury, preceding the activation of TGF-β/Smad signalling, suggesting that MMP-14 may regulate the bioactivity of TGF-β1 and stabilise developed blood vessels^21^. MMP-14 has been shown to control MSCs differentiation^19^ and MMP-14 deficiency impairs MSCs invasion^62^. Therefore, our findings suggest that MMP-14 may be involved in the recruitment and migration of MSCs toward damaged tissues for remodelling. MMP-14 may also activate TGF-β via proteolysis of LTBP-1^21^, which in turn promotes Smad phosphorylation/activation.

## Conclusion

The findings presented here reveal a possible mechanism for vascular repair/remodelling via TGF-β signalling. The activated TGF-β1 recruits MSCs and promotes their proliferation and differentiation into neointimal cells, leading to ECM deposition. TGF-β1 also mobilises MSCs from the bone marrow to the peripheral blood by which MSCs migrate to sites of injury. Whether recruited MSCs contribute to the pathogenesis of vascular disorders remains unclear and needs further investigations. MMP-14 also participates in neointimal formation by releasing activated TGF-β1 and promoting MSC recruitment. However, the precise function of MMP-14 in the vascular repair remains to be investigated and further experimental designs may include effective outcomes of employing an MMP-14 inhibitor in a balloon-induced arterial injury model and of placing MMP-14 to a co-culture system of VSMC and MSC.

## Methods

All experiments were carried out in accordance with the approved guidelines of the Good Experimental Practices adopted by the Institute of Zoology, Chinese Academy of Sciences. Experimental protocols and animal handling procedures were approved by the Committee for Animal Experiments of the Institute of Zoology, Chinese Academy of Sciences, China. All the methods followed the approved protocol and were conducted in accordance with the approved guidelines.

### Drugs

SB431542 was purchased from Sigma-Aldrich (St. Louis, MO, USA) and rhTGF-β1 was purchased from R&D (R&D Systems, Minneapolis, MN, USA); stock solutions (20 mg/ml and 100 μg/ml, respectively) were prepared in dimethylsulfoxide (DMSO).

### Experimental animals

Sprague-Dawley rats were purchased from the Animal Center of Xinjiang Medical University, Xinjiang, China. All animal procedures were carried out in accordance with the U.S. National Institute of Health guidelines. Rats were housed in polycarbonate cages containing sterile wood chips and maintained in sterile Trexler Plastic film isolators (Fengshi Laboratory Animal Equipment, Jiangsu, China) at 23 °C–26 ^o^C and a relative humidity of 50% ± 5% on a 12:12-hlight/dark cycle. Male rates (n = 120, weight: 350–400 g) were randomly divided into three treatment groups (n = 5 per group): vehicle-treated, SB-treated, and rhTGF-β1-treated. PTCA dilatation catheters (2.0/15 mm) were purchased from Thermo Fisher Scientific (Waltham, MA, USA). Surgical instruments used in this study were purchased from 66 vision (Suzhou, China).

### Rat model of Fogarty balloon-induced injury of the left carotid artery

Transluminal mechanical injury of the carotid artery was carried out according to a previously described protocol^63^. Briefly, rats were anaesthetised by intraperitoneal injection of chloral hydrate (3 ml/kg)and then immobilised on the operating floor with an incision made in the middle of the neck. The left carotid artery was subjected to blunt dissection, and the carotid artery and bifurcation were fully exposed. A Fogarty balloon (2.0/15 mm; Thermo Fisher Scientific) inflated with saline (2.0 atmospheres) was inserted towards the common carotid artery and then pulled back to the external carotid artery. This procedure was repeated three times to ensure complete endothelial denudation. Blood flow in the carotid artery was restored by releasing the arterial clamp placed in the proximal and distal portions. To block TGF-β1 signalling, SB431542 (0.2 mg/rat) or rhTGF-β1 (40 μg/rat) was injected into the respective groups every 48 h, while rats in the vehicle-treated group were administered 50% DMSO in 0.9% saline. After the operation, animals were treated with penicillin to prevent infection. Rats were euthanised at 0 h 8 h, 1 day, 3, 7, 14, 21, and 28 days after injury (n = 5). Bone marrow was collected by flushing 5 ml 0.1% bovine serum albumin (BSA) in phosphate-buffered saline (PBS) through the femurs and collecting 2 ml peripheral blood by cardiac puncture.

### Haematoxylin and eosin staining

Vessels were dissected and embedded in paraffin after fixation in 10% neutral formaldehyde. Sections (3 μm thick) were stained with haematoxylin and eosin. I/M ratios were calculated to assess neointimal formation 3, 7, 14, 21, and 28 days after injury. The ratios were determined with a computer-based morphological analysis system (Image Pro Plus 6.0; Olympus, Tokyo, Japan).

### Immunohistochemistry and immunofluorescence

Paraffin-embedded arteries were cut into 3-μm sections for immunohistochemical analysis. The EnVision Systems two-step immunohistochemical kit and diaminobenzidine enhancer (Dako,Glostrup, Denmark) were used to detect proteins of interest. PBS was used instead of the primary antibody as a negative control. Sections were deparaffinised with xylene, rehydrated in a graded series of alcohol, and quenched with 3% hydrogen peroxide. Heat-induced antigen retrieval was carried out in citrate buffer (pH 6.0) or in EDTA (pH 9.0). Sections were incubated overnight at 4 °C with the following primary antibodies: rabbit anti-TGF-β1 (sc-146; Santa Cruz Biotechnology, USA, 1:200 dilution), a rabbit anti-TβRI (sc-398;Santa Cruz Biotechnology, USA, 1:200 dilution), rabbit anti-p-Samd2/3 (sc-11769; Santa Cruz Biotechnology, USA, 1:800 dilution), and mouse anti-Vimentin (sc-32322; Santa Cruz Biotechnology, USA, 1:800 dilution); rabbit anti-MMP-14 (ab51074; Abcam, UK, 1:200 dilution), a mouse anti-Calponin (M-3556, Dako, Denmark, 1:800 dilution), a mouse anti-Vimentin (sc-32322, Santa Cruz Biotechnology, USA, 1:800 dilution), a rabbit anti-SMA (ab51074; Abcam, UK, 1:200 dilution), rabbit anti-CD31 (ab28364, Abcam, UK, 1:600 dilution), and rabbit anti-CD34 (ab8536; Abcam, UK, 1:400 dilution) mouse anti-Ki-67 (M-724801, Dako, Denmark, 1:600 dilution). Sections were washed with PBS and then incubated with secondary antibodies at 37 °C; immunoreactivity was visualised by incubation with diaminobenzidinetetrahydrochloride, followed by counterstaining with haematoxylin. immunofluorescence analysis of Nestin and CD29 expression was carried out as previously described. After blocking in 0.5% bull serum albumin for 30 min, sections were incubated with the following primary antibodies:rabbit anti-Nestin and rabbit anti-CD29 (EP1041Y and SP103; Abcam, UK, 1:100 dilution), followed by incubation with fluorescein isothiocyanate (FITC)-conjugated secondary antibodies (Zhongshan Golden Bridge Biotechnology Co., Beijing, China). Nuclei were counterstained with DAPI (Sigma-Aldrich, USA) and sections were visualised by confocal microscopy (FluoviewFV300; Olympus). Immunostaining was evaluated by three experienced pathologists who were blinded to the samples, and any differences in interpretation were resolved by consensus. Brownish-yellow staining appearing at the cell membrane or in the cytoplasm and/or nucleus was considered as a positive signal.

### Western blot analysis

The protocol for western blot analysis has been previously described^14^. Frozen carotid artery segments were washed with ice-cold PBS. Radioimmunoprecipitation assay buffer with phenylmethanesulphonyl fluoride (1:100 dilution) was added to the artery. Samples were centrifuged at 4 °C and 12,000 rpm for 20 min, and the supernatant was collected and total protein concentration was determined using a bicinchoninic protein assay kit (Pierce, Rockford, IL, USA). Proteins were mixed with loading buffer and heated at 100°C. Two hundred micrograms of protein extract from each sample was loaded onto 10% SDS-PAGE (15% SDS-PAGE for TGF-β1, 6% SDS-PAGE for Nestin and 10%SDS-PAGE for other proteins) (Bio-Rad, California, USA). Samples were transferred to polyvinyl difluoride membranes (Solarbio, Beijing, China), which were blocked with 5% nonfat milk in Tris-buffered saline with 0.1% Tween-20 for 1–2 h at room temperature and then incubated with overnight at 4 °C with the following primary antibodies: rabbit anti-TGF-β1 (sc-146, Santa Cruz Biotechnology, USA), rabbit anti-TβRI (sc-398, Santa Cruz Biotechnology, USA), rabbit anti-p-Samd2/3 (sc-11769, Santa Cruz Biotechnology, USA), rabbit anti-Smad2/3 (sc-8332;Santa Cruz Biotechnology, USA);rabbit anti-MMP-14 (ab51074, Abcam, UK), mouse anti-Calponin (M-3556, Dako, Denmark), and a rabbit anti-Nestin (N5413; Sigma, USA). This was followed by incubation with horseradish peroxidase-conjugated sheep anti-mouse (1:20000) or anti-rabbit (1:5000) secondary antibody for 2 h at room temperature. Bands were detected by enhanced chemiluminescence and exposure to X-ray films. Bands were scanned and analysed with Imagesoftware (National Institutes of Health, Bethesda, MD, USA). β-Actin served as a loading control.

### Flow cytometry

Blood samples were collected from rats by cardiac puncture. After removing red blood cells with lymphocyte-isolating solution (Solarbio Systems, Beijing, China), bone marrow samples were collected from the femur of rats. Cells were washed with 0.1% BSA in PBS, and 1 × 10^6^ cells/ml were incubated with antibodies for 30 min at 37 °C in a dark room. Sca1^+^CD29^+^CD11b/c^−^CD45^−^cells were considered as the MSCs fraction. Cells were stained with the following combination of antibodies against cell surface markers: phycoerythrin-conjugated anti-Sca-1 (D7, Bio-Legend), FITC-conjugated anti-CD29 (HMβ1-1, Bio-Legend), peridinin chlorophyll protein complex-conjugated anti-CD45 (OX-1, Bio-Legend), and allophycocyanin-conjugated anti-CD11b/c (OX-42) antibodies were used. Cells were sorted using a FACS Calibur flow cytometer and CellQuest software (Becton Dickinson, Franklin Lakes, NJ, USA).

### Statistical analysis

Data were analysed using SPSS v.17.0 software (IBM Corp., Armonk, NY, USA). Quantitative data are presented as mean ± standard deviation of at least three separate experiments. Drug doses were selected based on single factor analysis of variance. The Student’s *t*-test was used to evaluate differences in grey value. Statistical significance was set at P < 0.05.

## Additional Information

**How to cite this article**: Zhao, W. *et al.* Effect of TGF-β1 on the Migration and Recruitment of Mesenchymal Stem Cells after Vascular Balloon Injury: Involvement of Matrix Metalloproteinase-14. *Sci. Rep.* **6**, 21176; doi: 10.1038/srep21176 (2016).

## Supplementary Material

Supplementary Information
