# Supplementary material for: Effect of TGF-β1 on the Migration and Recruitment of Mesenchymal Stem Cells after Vascular Balloon Injury: Involvement of Matrix Metalloproteinase-14
Source: Sci Rep. 2016 Feb 16;6:21176. doi: 10.1038/srep21176 (PMC4754777; doi:10.1038/srep21176)
Supplement: Supplementary Information [file srep21176-s1.pdf]

# Effect of TGF-β1 on the Migration and Recruitment of Mesenchymal Stem Cells after Vascular Balloon Injury: Involvement of Matrix Metalloproteinase-14

Wei Zhao[#1], Chengyan Wang[#1], Ruixue Liu[1], Cuilei Wei[1], Juncang Duan[2], Kejian Liu[2], Shugang Li[3], Hong Zou[1], Jin Zhao[1], Lianghai Wang[1], Yan Qi[1], Weihua Liang[1], Jinfang Jiang[1], Wenjie Zhang[1], Lijuan Pang*[1], Feng Li*[1]

[1]Department of Pathology and Key Laboratory of Xinjiang Endemic and Ethnic Diseases (Ministry of Education), Shihezi University School of Medicine, 59 North 2nd Road, Shihezi, Xinjiang 832002, China

[2]Department of Cardiology, the First Affiliated Hospital of Shihezi city, 45 North 3rd Road, Shihezi, Xinjiang 832002, China

[3]Department of Public Health, Shihezi University School of Medicine, 59 North 2nd Road, Shihezi, Xinjiang 832002, China

#Co-first authors:

**Wei Zhao**, E-mail:18599046455@163.com

**Chengyan Wang**, E-mail: 513935508@qq.com

Both authors contributed equally to this work

*Corresponding authors:

**Lijuan Pang**, MD, PhD E-mail: ocean123456@163.com
**Feng Li**, MD, PhD E-mail: lifeng7855@126.comFax: +86-0993-205-713

**Supplement Figure Legends**

**Supplement Figure 1. Immunohistochemical analysis of Ki-67, CD31, and CD34 expression in injured arteries.**

**A–C:** Representative images of Ki-67(A), CD31 (B), and CD34 (C) expression. Scale bar, 50 μm.

**Supplement Figure 2. Immunohistochemical analysis of Calponin, SMA, and Vimentin expression in injured arteries.**

A. **C:** Representative images of Calponin (A), SMA (B), and Vimentin (C). Scale bar, 50 μm.

**Supplement Figure 3. MMP-14 expression after arterial injury.**

Representative images of MMP-14 expression in injured arteries, as detected by immunohistochemistry.Scale bar, 50 μm.

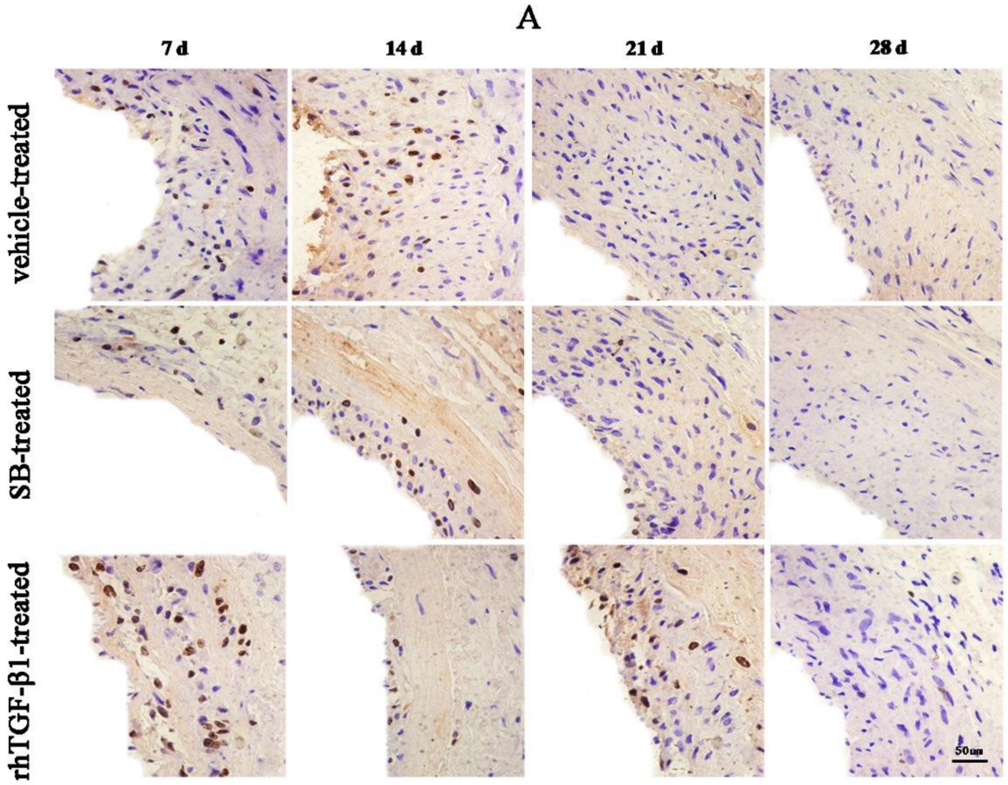


**Supplement Fig. 1A (Ki-67)**

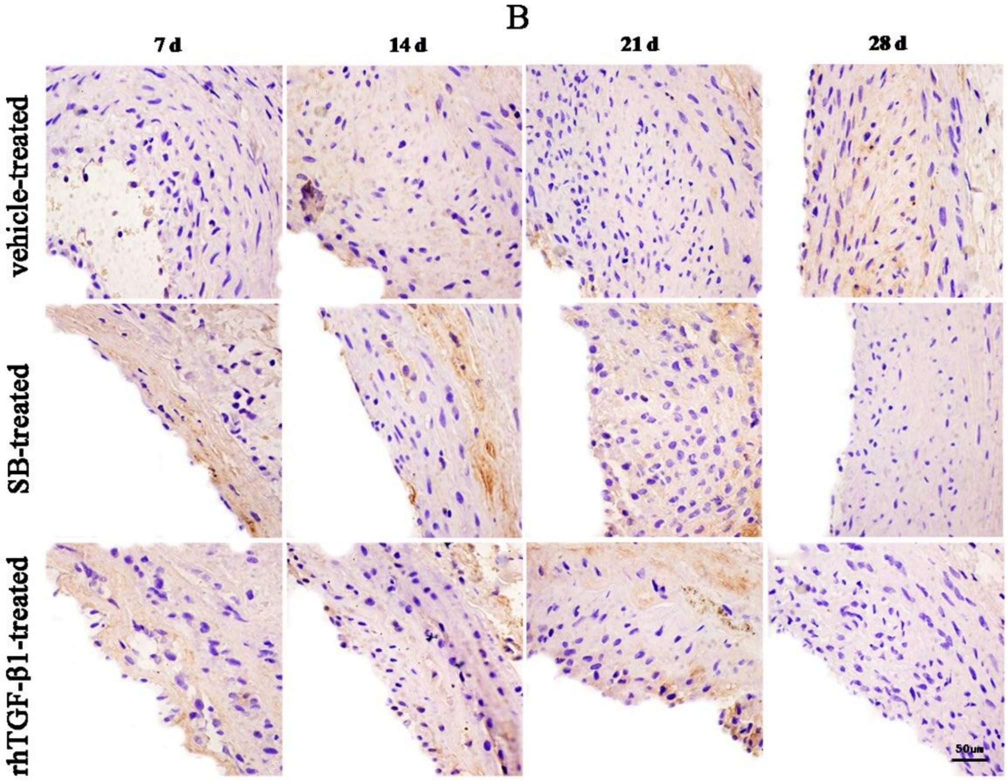


**Supplement Fig. 1B (CD31)**

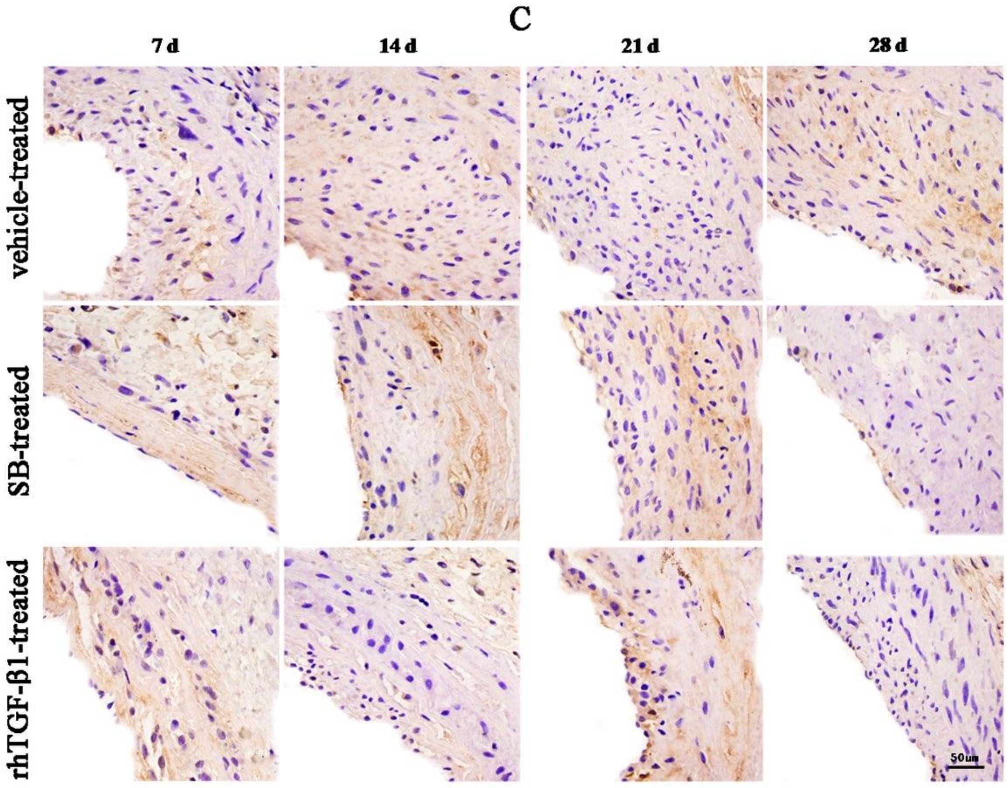


**Supplement Fig. 1C (CD34)**

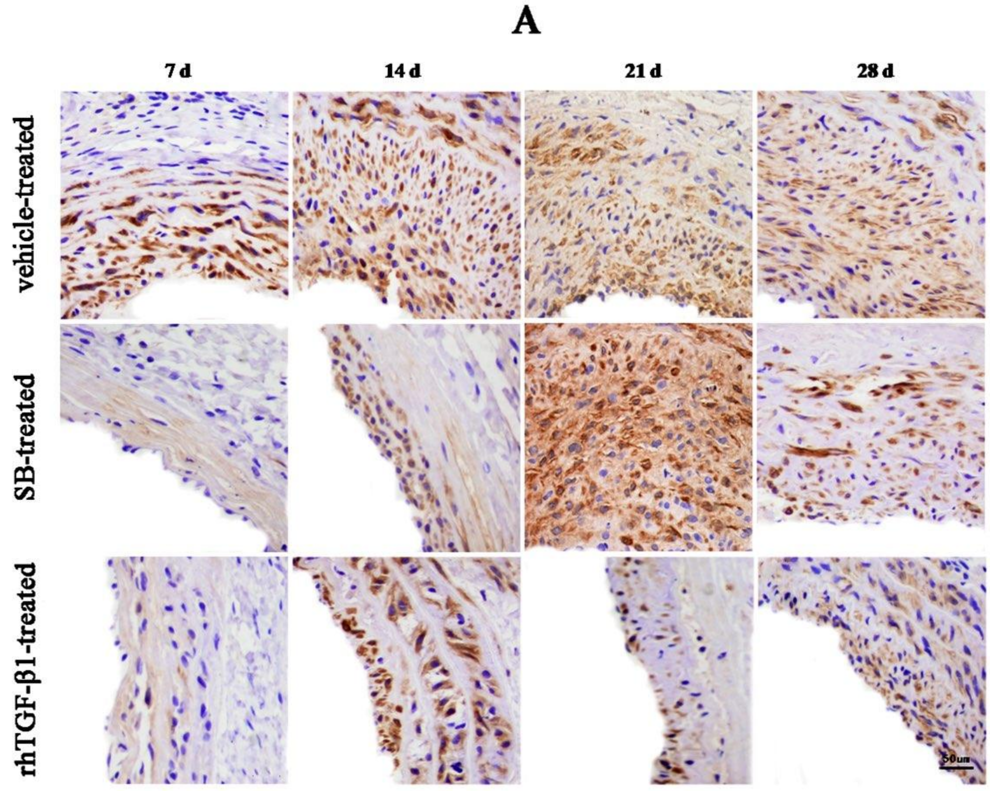


**Supplement Fig. 2A (Calponin)**

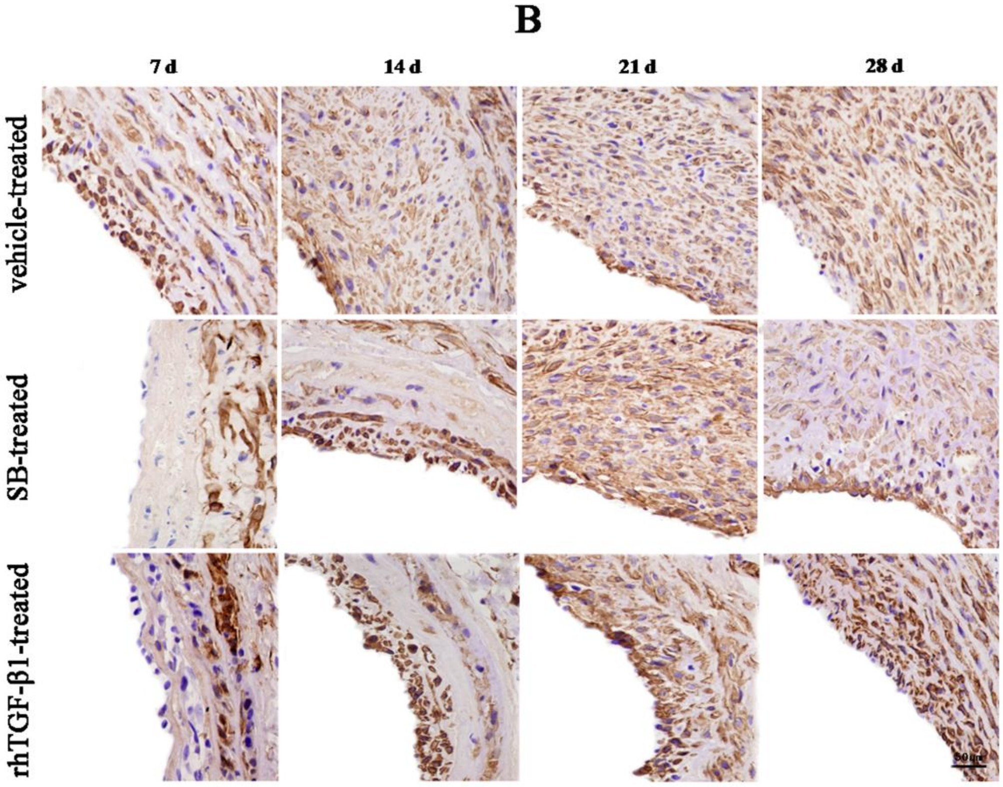


**Supplement Fig. 2B (SMA)**

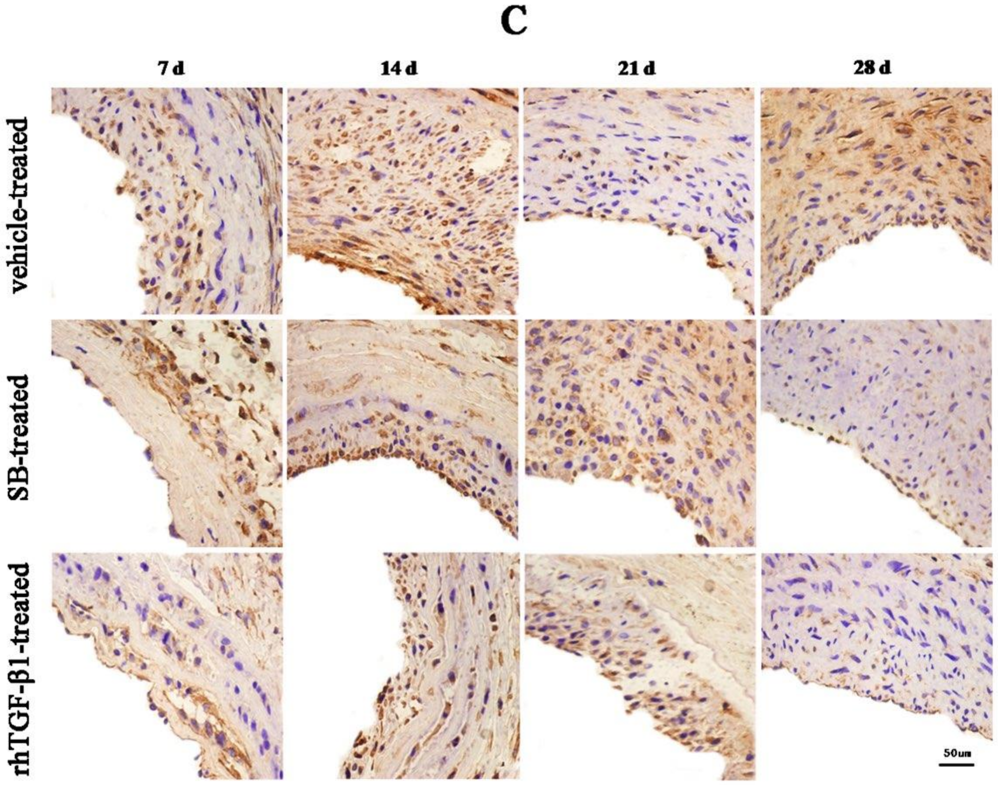


**Supplement Fig. 2C (Vimentin)**

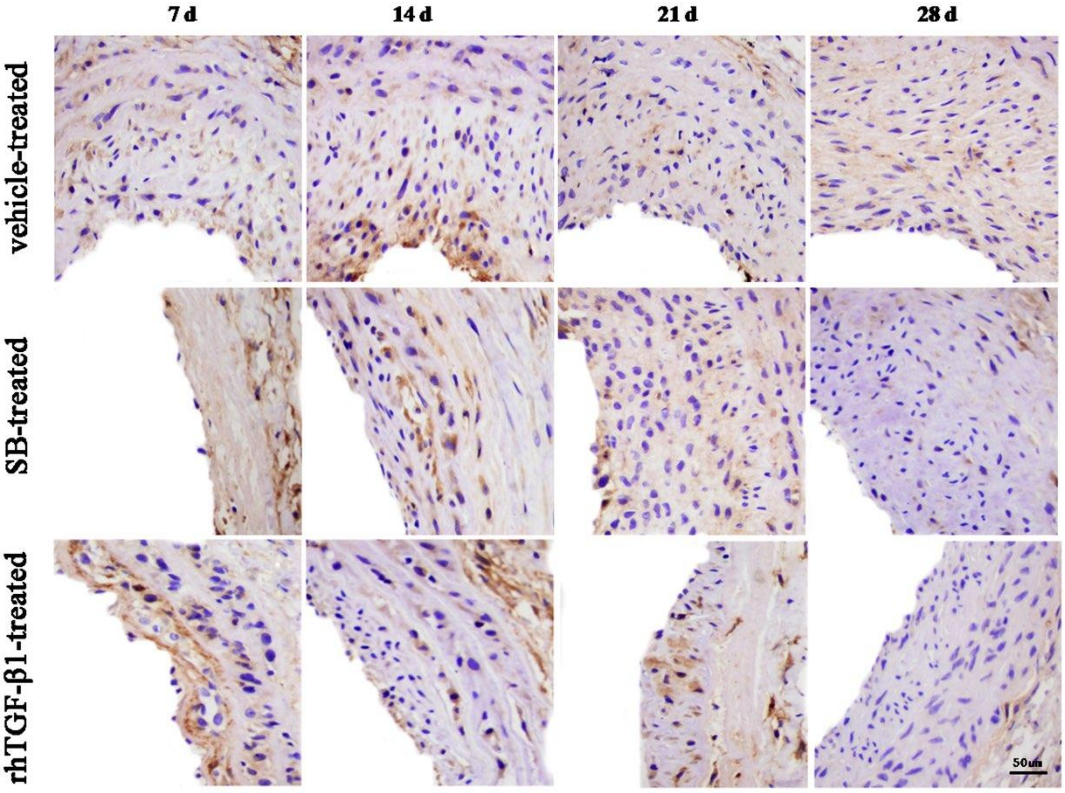


**Supplement Fig. 3 (MMP-14)**

**Figure 2A**

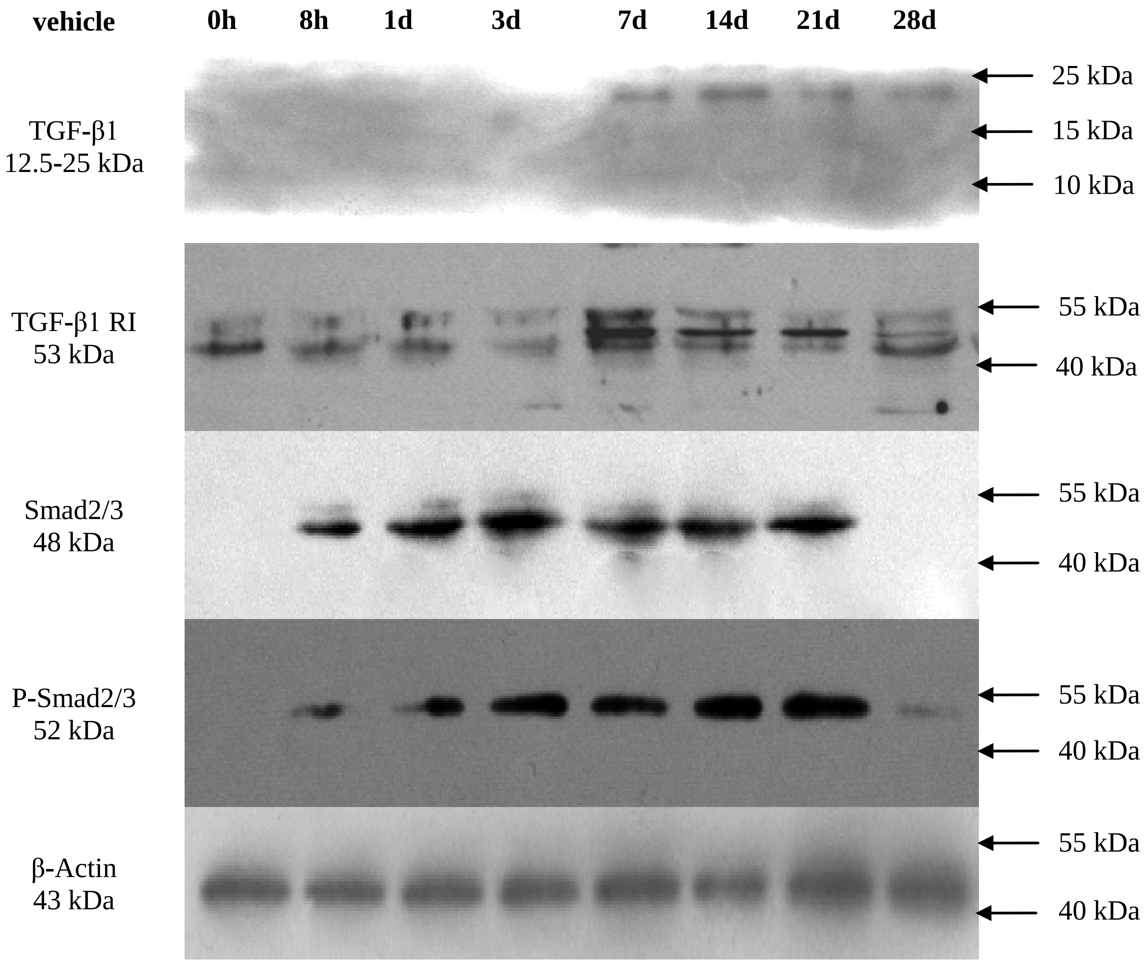

vehicle
0h
8h
1d
3d
7d
14d
21d
28d
TGF-β1
12.5-25 kDa
25 kDa
15 kDa
10 kDa
TGF-β1 RI
53 kDa
55 kDa
40 kDa
Smad2/3
48 kDa
55 kDa
40 kDa
P-Smad2/3
52 kDa
55 kDa
40 kDa
β-Actin
43 kDa
55 kDa
40 kDa

**Figure 2B**

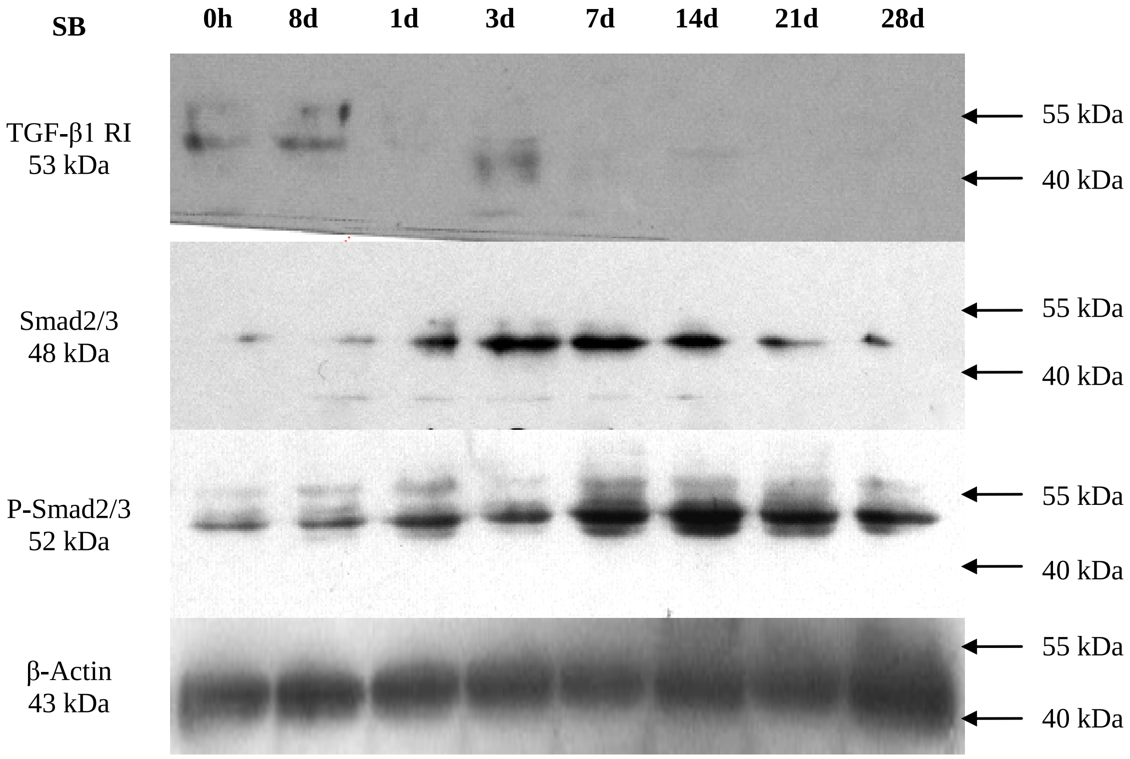
SB
0h
8d
1d
3d
7d
14d
21d
28d
TGF-β1 RI
53 kDa
Smad2/3
48 kDa
P-Smad2/3
52 kDa
β-Actin
43 kDa
55 kDa
40 kDa
55 kDa
40 kDa
55 kDa
40 kDa
55 kDa
40 kDa

**Figure 2C**

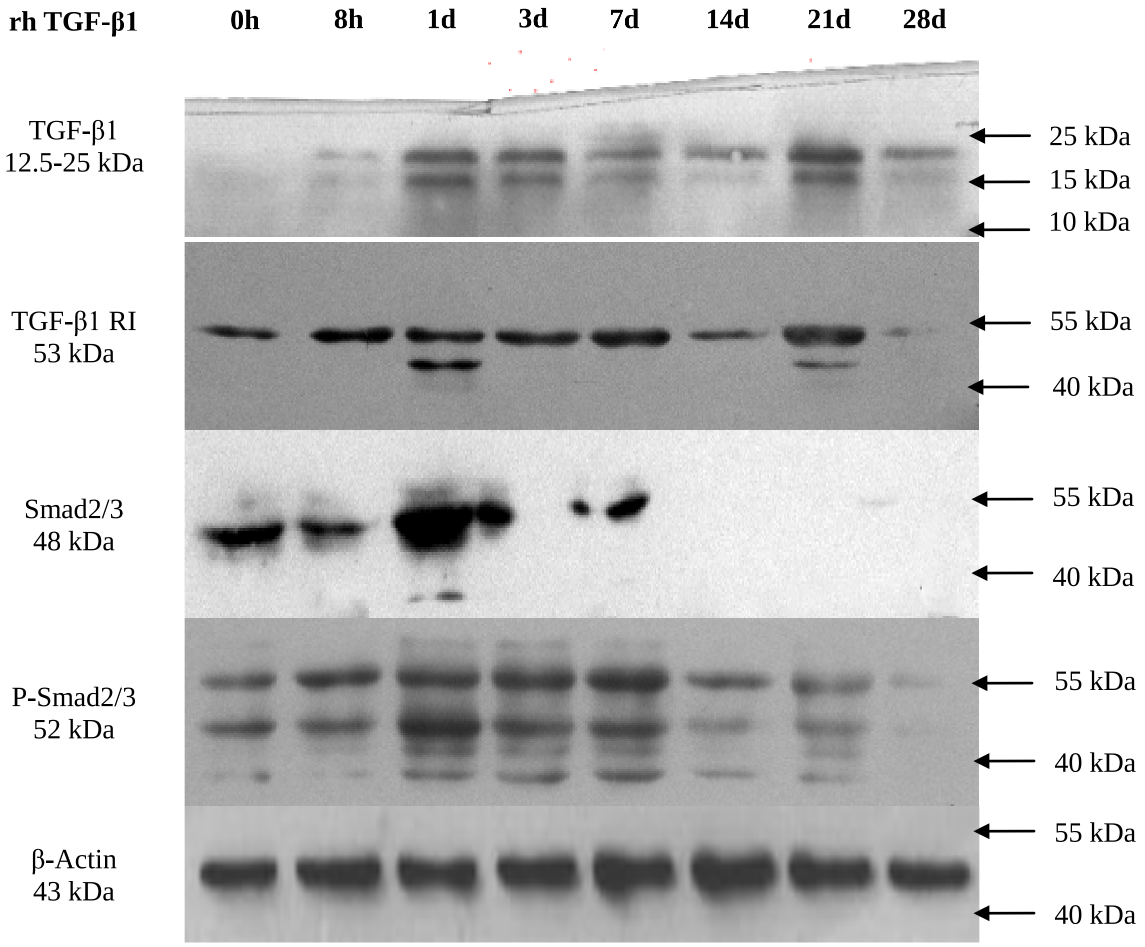

rh TGF-β1
0h
8h
1d
3d
7d
14d
21d
28d
TGF-β1
12.5-25 kDa
25 kDa
15 kDa
10 kDa
TGF-β1 RI
53 kDa
55 kDa
40 kDa
Smad2/3
48 kDa
55 kDa
40 kDa
P-Smad2/3
52 kDa
55 kDa
40 kDa
β-Actin
43 kDa
55 kDa
40 kDa

## Figure 5

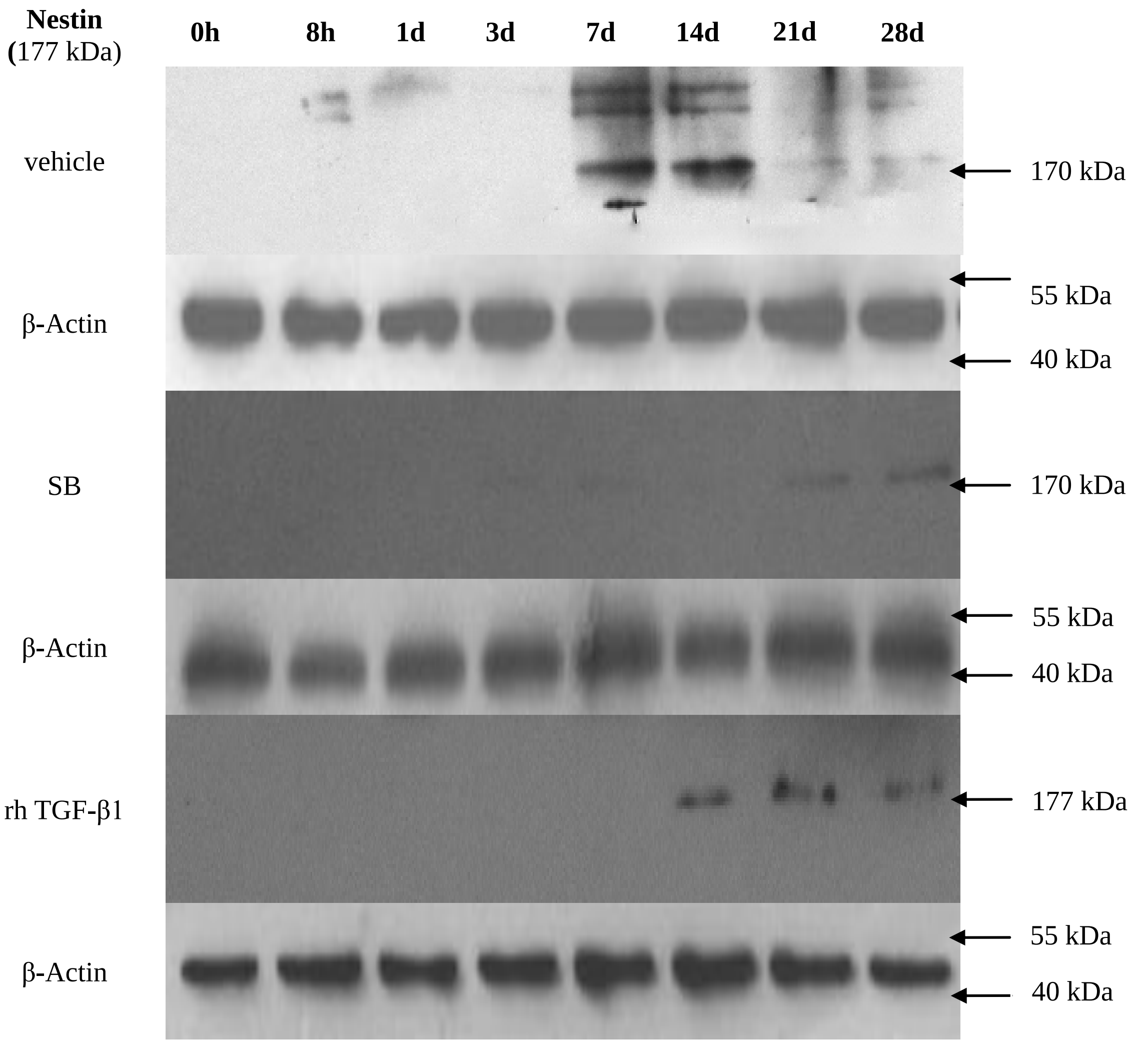
Nestin
(177 kDa)
0h
8h
1d
3d
7d
14d
21d
28d
vehicle
170 kDa
β-Actin
55 kDa
40 kDa
SB
170 kDa
β-Actin
55 kDa
40 kDa
rh TGF-β1
177 kDa
β-Actin
55 kDa
40 kDa

**Figure 6A**

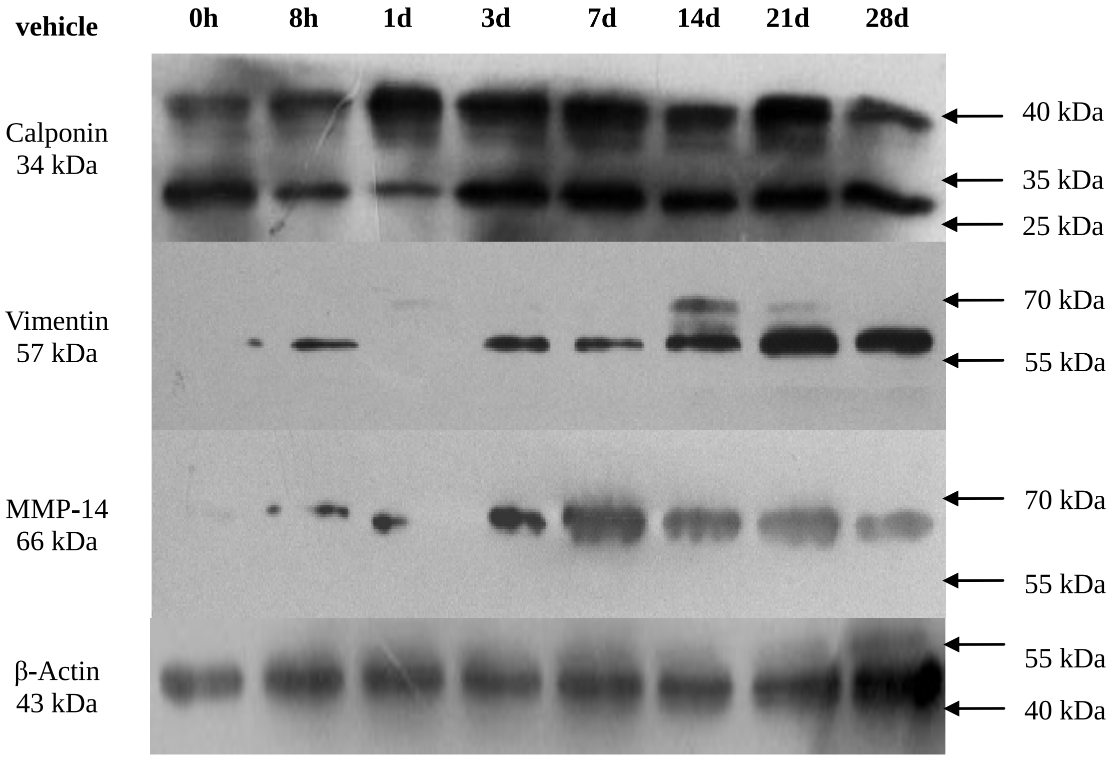

vehicle
0h
8h
1d
3d
7d
14d
21d
28d
Calponin
34 kDa
Vimentin
57 kDa
MMP-14
66 kDa
β-Actin
43 kDa
40 kDa
35 kDa
25 kDa
70 kDa
55 kDa
70 kDa
55 kDa
55 kDa
40 kDa

**Figure 6B**

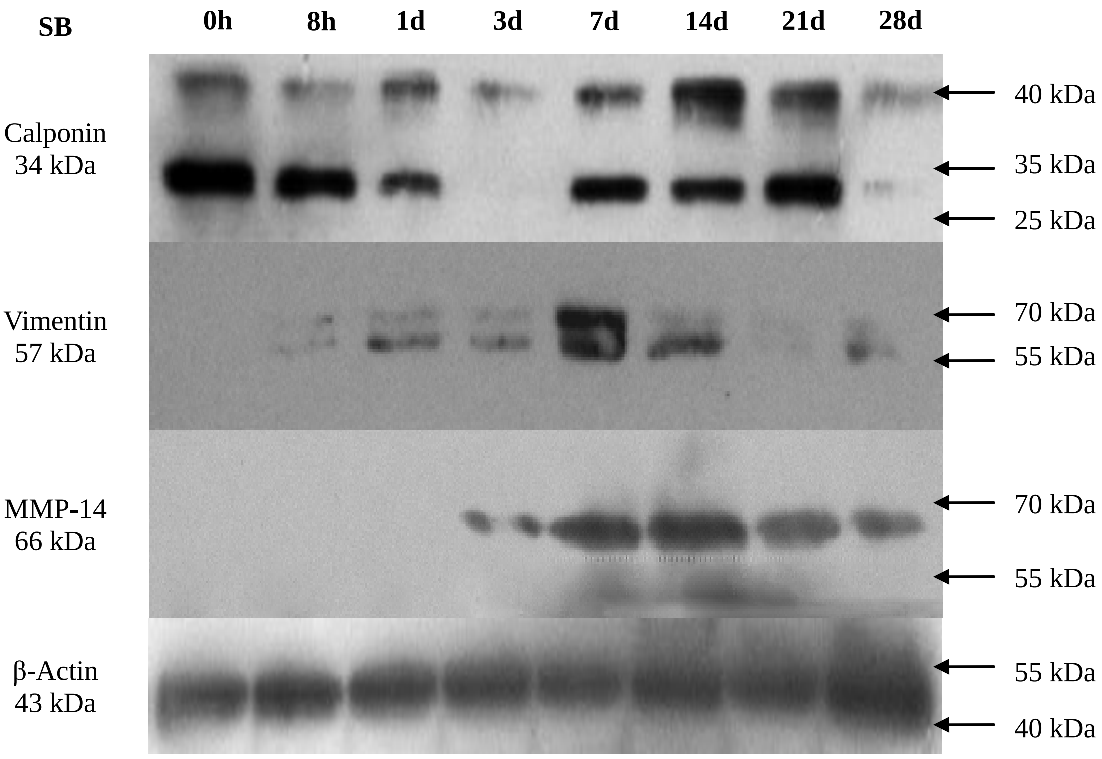
SB
0h
8h
1d
3d
7d
14d
21d
28d
Calponin
34 kDa
Vimentin
57 kDa
MMP-14
66 kDa
β-Actin
43 kDa
40 kDa
35 kDa
25 kDa
70 kDa
55 kDa
70 kDa
55 kDa
55 kDa
40 kDa

**Figure 6C**

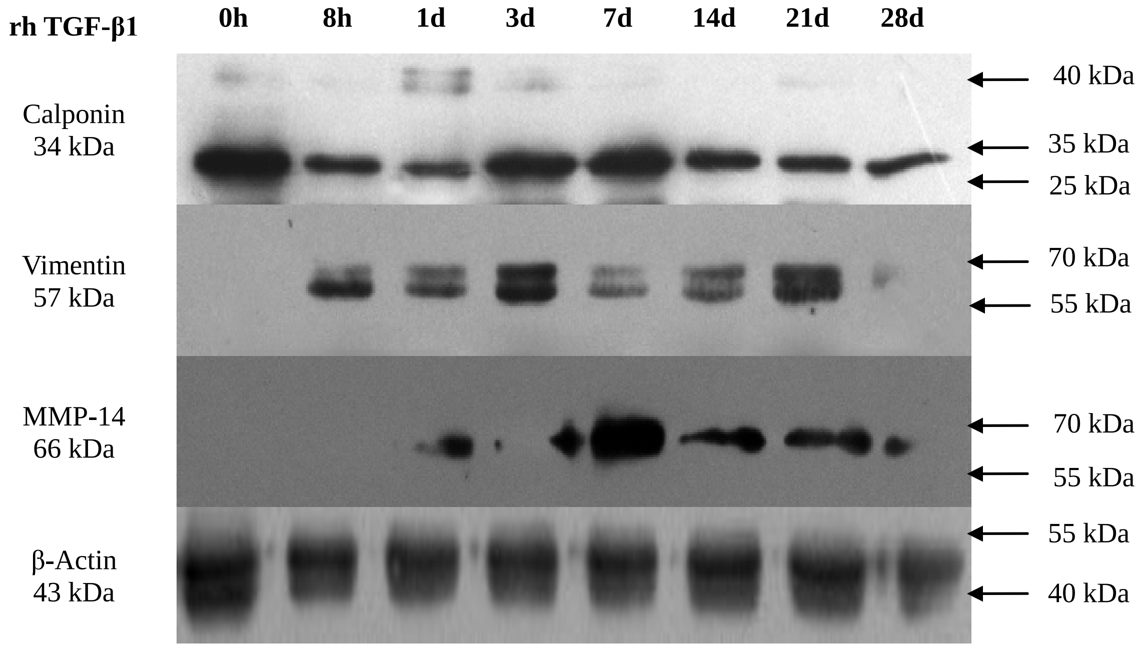
rh TGF-β1
0h
8h
1d
3d
7d
14d
21d
28d
Calponin
34 kDa
Vimentin
57 kDa
MMP-14
66 kDa
β-Actin
43 kDa
40 kDa
35 kDa
25 kDa
70 kDa
55 kDa
70 kDa
55 kDa
55 kDa
40 kDa
